# Urinary Beta-2-Microglobulin and Late Nephrotoxicity in Childhood Cancer Survivors

**DOI:** 10.3390/jcm10225279

**Published:** 2021-11-13

**Authors:** Eryk Latoch, Katarzyna Konończuk, Katarzyna Taranta-Janusz, Katarzyna Muszyńska-Rosłan, Magdalena Sawicka, Anna Wasilewska, Maryna Krawczuk-Rybak

**Affiliations:** 1 Department of Pediatric Oncology and Hematology, Medical University of Bialystok, 15-274 Białystok, Poland; kononczukk@gmail.com (K.K.); kmroslan@post.pl (K.M.-R.); rybak@umb.edu.pl (M.K.-R.); 2 Department of Pediatrics and Nephrology, Medical University of Bialystok, 15-274 Białystok, Poland; katarzyna.taranta@wp.pl (K.T.-J.); annwasil@interia.pl (A.W.); 3 Department of Analysis and Bioanalysis of Medicines, Medical University of Bialystok, 15-089 Białystok, Poland; magdalena.sawicka@umb.edu.pl

**Keywords:** B2M, cancer, CCS, children, CKD, chronic kidney disease, nephropathies, renal toxicity

## Abstract

The objectives of this study were to evaluate urinary beta-2-microglobulin (β2M) levels in long-term childhood cancer survivors and to establish its association with anticancer drug-induced nephrotoxicity. The study consisted of 165 childhood cancer survivors (CCS) who were in continuous complete remission. We reported that CCS had a significantly higher level of β2M (*p* < 0.001) and β2M/Cr. ratio (*p* < 0.05) than healthy peers. Among all participants, 24 (14.5%) had decreased eGFR (<90 mL/min/1.73 m^2^). A significant positive correlation between β2M/Cr. ratio and body mass index (coef. 14.48, *p* = 0.046) was found. Furthermore, higher levels of urinary β2M were detected among CCS with a longer follow-up time (over 5 years) after treatment. Subjects with decreased eGFR showed statistically higher urinary β2M levels (20.06 ± 21.56 ng/mL vs. 8.55 ± 3.65 ng/mL, *p* = 0.007) compared with the healthy peers. Twelve survivors (7.2%) presented hyperfiltration and they had higher urinary β2M levels than CCS with normal glomerular filtration (46.33 ± 93.11 vs. 8.55 ± 3.65 ng/mL, *p* = 0.029). This study did not reveal an association between potential treatment-related risk factors such as chemotherapy, surgery, radiotherapy, and the urinary β2M level. The relationship between treatment with abdominal radiotherapy and reduced eGFR was confirmed (*p* < 0.05). We demonstrated that urinary beta-2-microglobulin may play a role in the subtle kidney injury in childhood cancer survivors; however, the treatment-related factors affecting the β2M level remain unknown. Further prospective studies with a longer follow-up time are needed to confirm the utility of urinary β2M and its role as a non-invasive biomarker of renal dysfunction.

## 1. Introduction

Improvements in the diagnosis and treatment of pediatric cancers have now resulted in significantly higher survival rates. However, childhood cancer survivors (CCS) are at greater risk of experiencing many treatment-related adverse effects that substantially affect later quality of life [1,2]. Nephrotoxicity is one of the most common late sequelae with prevalence ranging up to 80% based on the population studied [3]. There are many known causes of renal damage among CCS. These may result from the multimodal therapy (chemotherapy, radiotherapy, immunotherapy, and surgery), supportive treatment used (aminoglycoside antibiotics, diuretics, or antifungal agents), and the cancer itself—by tumor infiltration, disruption of glomerular and tubular development, acute tumor lysis syndrome, or urinary tract obstruction. The most nephrotoxic cytostatics include platinum-based drugs such as cisplatin and carboplatin, as well as ifosphamide, cyclophosphamide, and methotrexate. Overall, CCS have a nine-fold increased risk of developing chronic nephrotoxicity compared to their siblings, according to one of the largest studies available [4].

The extent of renal impairment can vary and depends on many factors. In some cases, the only sign of kidney damage is renal hypertension or Fanconi syndrome, while others develop chronic kidney disease (CKD) from mild damage to complete kidney failure at various ages in life. Based on available methods, it remains difficult to predict which patients will develop kidney disease after anticancer treatment. The primary parameter assessing renal function is estimated glomerular filtration rate (eGFR) based on creatinine level. However, there are many limitations affecting its level which include age, sex, race, muscle mass, diet, hydration status, physical activity, and medications taken regularly. New markers are being sought to identify individuals with early renal function impairment before it is clinically detectable by standard techniques. In recent years, there has been an increasing number of studies on the role of beta-2-microglobulin (β2M) in the development of acute kidney injury (AKI) and CKD. Some of them, but not all, reported its elevated levels following treatment with nephrotoxic drugs [5,6]. Still little is known about the association of β2M with late drug-induced nephrotoxicity in childhood cancer survivors.

Beta-2-microglobulin is a small protein (18 kDa) encoded by gen located on chromosome 15. In healthy subjects it is filtered by glomeruli and subsequently reabsorbed in the proximal tubule of the nephron, resulting in minimal urine concentration [7,8]. Tubular injury leads to increased urinary β2M levels due to impaired reabsorption. Furthermore, unlike creatinine, β2M levels do not depend on muscle mass, which makes it a potential candidate biomarker of kidney damage [9].

The aims of this study were to evaluate urinary beta-2-microglobulin levels in long-term childhood cancer survivors and determine its relationship with the type of anticancer treatment used.

## 2. Materials and Methods

The study included 165 childhood cancer survivors (80 males and 85 females) visiting the follow-up outpatients’ clinic at the Department of Pediatric Oncology and Hematology, Medical University of Bialystok (Poland). All participants were in complete first continuous remission. Exclusion criteria included: congenital anomalies of kidney or urinary tract, current infection, relapse of cancer. All the children were treated according to the international protocols in line with the diagnosis, approved by the Polish Pediatric Leukemia and Lymphoma Group, and Polish Pediatric Solid Tumors Group. Written informed consent was obtained from the participants or their parents/guardians. The study was approved by the Ethical Committee of the Medical University of Bialystok in accordance with the Declaration of Helsinki (permission number: R-I-002/62/2018).

Clinical history, including demographic information, comorbidities, anticancer treatment used in each patient, especially cumulative dosage of nephrotoxic drugs (cyclophosphamide, ifosfamide, cisplatin, methotrexate), and abdominal radiotherapy was obtained from the CCS database. All the participants underwent a clinical examination and anthropometric measurements. Body mass index (BMI) was calculated as weight in kilograms divided by height in squared meters (kg/m^2^). Blood pressure was measured using a standardized sphygmomanometer (performed three times at 1–2 min intervals); before the measurement, the participant rested peacefully for five minutes. Hypertension was defined as mean systolic blood pressure (SBP) and/or diastolic blood pressure (DBP) level ≥ 95th percentile adjusted for age, sex, and height [10]. The control group consisted of 50 healthy peers (27 female) without history of urinary tract infections, with a pair of normal kidneys, who were offspring of the departments’ employees. 

All participants had an abdominal ultrasound to assess the kidney and urinary tract performed by a qualified radiologist. After a 12 h night, fasting peripheral blood was collected from each participant for routine laboratory testing. Clean catch urine samples were stored at −80 °C for further analysis. The serum creatinine level was measured by enzymatic method, and the estimated glomerular filtration rate (mL/min/1.73 m^2^) was calculated using the updated Schwartz formula: eGFR = 0.413 × (height in cm/serum cr. in mg/dL). Urinary beta-2-microglobulin level was measured using commercial immunoassays (R&D SYSTEMS a bio-techne brand, Quantikine^®^ ELISA, Minneapolis, USA) according to the instructions for the ELISA kit. The tested urine biomarkers were calculated per milligram urine creatinine (cr.) in order to avoid the effect of urine dilution (β2M/cr. ratio). Urine albumin concentration was determined by Lowry’s method. Subjects with a urinary albumin/creatinine ratio between 30 to 300 μg/mg were considered to have albuminuria. The stages of CKD were classified according to Kidney Disease: Improving Global Outcomes (KDIGO) guidelines, defined by structural or functional abnormalities of the kidney for ≥3 months, with or without decreased eGFR or eGFR < 60 mL/min/1.73 m² for ≥3 months, with or without kidney damage [11]. Glomerular hyperfiltration was define as eGFR higher than 175 mL/min/1.73 m^2^ [12]. 

Statistical analysis. Normal distribution was tested by using the Shapiro–Wilk test. Mean and standard deviation (SD) or median (Me) and interquartile range (IQR) were used to presented data. The *t*-Student test or Mann–Whitney U test were applied to compare independent variables depending on normal distribution. Univariate analysis of variance was performed using ANOVA, and post hoc analysis using Tukey’s test. The correlations between β2M and urine creatinine, blood pressure and cumulative dose of cytostatics used were calculated by Spearman or Pearson correlation coefficients. The STATA 12.1 version (StatCorp, College Station, TX, USA) was used to perform statistical analysis, and statistical significance was determined at 0.05.

## 3. Results

The clinical characteristics of the childhood cancer survivors (CCS) are presented in Table 1. The mean age at the time of diagnosis was 5.31 ± 4.16 years, while the mean time from treatment cessation to follow-up was 7.01 ± 5.28 years. The study group did not differ in age and sex from the control group.

Childhood cancer survivors revealed increased serum creatinine levels (0.60 ± 0.22 mg/dL vs. 0.52 ± 0.14 mg/dL, *p* = 0.035) as well as urine creatinine levels (131.24 ± 74.62 mg/L vs. 92.25 ± 45.97 mg/L, *p* = 0.001) than the healthy peers. However, we did not observe any difference in the eGFR (122.37 ± 34.93 mL/min/1.73 m^2^ vs. 124.51 ± 38.49 mL/min/1.73 m^2^, *p* = 0.792). 

**Figure 1:**
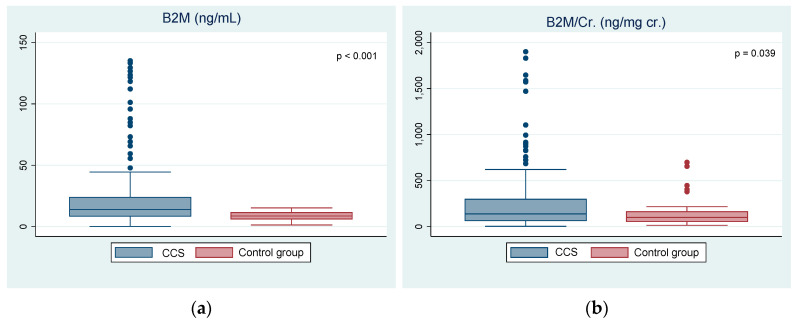
Comparison of (**a**) the urinary β2M (beta-2-microglobulin) and (**b**) the urinary β2M/Cr. ratio (beta-2-microglobulin/creatinine) between the childhood cancer survivors (CCS) and the control group.

The study group presented a significantly higher concentration of β2M (33.64 ± 59.53 ng/mL vs. 8.55 ± 3.65 ng/mL, *p* < 0.001) and β2M/Cr. ratio (277.96 ± 405.67 ng/mg cr. vs. 140.98 ± 144.09 ng/mg cr., *p* = 0.039) compared to the control group (Figure 1). The summary of the biochemical parameters is shown in Table 2.

We found no differences in β2M (33.43 ± 57.72 ng/mL vs. 33.86 ± 61.77 ng/mL, *p* = 0.727) and β2M/Cr. ratio (283.89 ± 438.08 ng/mg cr. vs. 271.65 ± 370.81 ng/mg cr., *p* = 0.965) concentrations according to sex. Both genders had significantly higher values of urine creatinine (female: 128.81 ± 71.87 mg/L vs. 96.76 ± 49.38 mg/L, *p* = 0.045; male: 133.82 ± 77.81 mg/L vs. 86.96 ± 42.08 mg/L, *p* = 0.008) and urinary β2M (female: 33.43 ± 57.72 ng/mL vs. 8.05 ± 3.64 ng/mL, *p* < 0.001; male: 33.86 ± 61.77 ng/mL vs. 9.13 ± 3.65 ng/mL, *p* = 0.001) when compared to the control group, but no gender differences were found within the study group. Of note, HSCT treatment had no effect on urinary β2M levels (*p* < 0.05).

Twenty-four of the study participants (14.5%) presented eGFR below 90 mL/min/1.73 m^2^ of which only one had eGFR below 60 mL/min/1.73 m^2^. Notably, before starting the treatment, eGFR was within normal range in all participants. The subgroup of subjects with reduced eGFR did not reveal any differences in β2M level and β2M/Cr. ratio in comparison to the normal eGFR subgroup. The analysis of patients with decreased eGFR compared with the healthy peers showed statistically higher β2M levels (20.06 ± 21.56 ng/mL vs. 8.55 ± 3.65 ng/mL, *p* = 0.007) in CCS, but no correlations between both the β2M level and β2M/Cr. ratio and eGFR were found. Among the whole study group, there were twelve individuals (7.2%) who presented hyperfiltration. Compared to those with normal glomerular filtration, they showed higher urinary creatinine (144.34 ± 75.35 mg/dL vs. 92.25 ± 45.97 mg/dL, *p* = 0.009) and β2M (46.33 ± 93.11 ng/mL vs. 8.55 ± 3.65 ng/mL, *p* = 0.029) levels.

We also investigated the association between the type of cytostatics used, abdominal radiotherapy, and the number of patients with reduced eGFR (<90 mL/min/1.73 m^2^) in each group according to whether or not they were exposed to a particular nephrotoxic agent. Survivors who had radiation therapy in childhood were statistically more likely to have decreased eGFR at the time of the study compared to those who had never received RT (11% vs. 31%, *p* = 0.024). 

Furthermore, we divided the study group according to the time of cessation of treatment. There were 101 subjects (61.2%) over 5 years after the end of treatment with significantly higher levels of sCr (0.68 ± 0.21 ng/mL vs. 0.48 ± 0.19 ng/mL, *p* < 0.0001), uCr (148.18 ± 79.62 mg/dL vs. 104.50 ± 56.98 mg/dL, *p* < 0.001), β2M (44.09 ± 71.76 ng/mL vs. 17.14 ± 24.50 ng/mL, *p* = 0.0001), β2M/Cr. ratio (306.17 ± 74.87 ng/mg cr. vs. 233.42 ± 363.09 ng/mg cr., *p* = 0.049), and lower eGFR (117.02 ± 28.68 mL/min/1.73 m^2^ vs. 131.56 ± 42.37 mL/min/1.73 m^2^, *p* = 0.047) than participants who were under 5 years from the end of treatment at follow-up (Table 3.).

In the whole group, positive correlations between urine creatinine (*r* = 0.25, *p* = 0.001), systolic blood pressure (*r* = 0.20, *p* = 0.015), diastolic blood pressure (*r* = 0.26, *p* = 0.001), and β2M level were found; however, the strength of relationships was very weak (*r* < 0.3).

Participants were also divided based on their initial diagnosis and treatment as follows: leukemia and non-Hodgkin lymphoma (*n* = 105), Hodgkin lymphoma (*n* = 10), and solid tumors (*n* = 50). No significant differences in eGFR (*p* = 0.149), β2M level (*p* = 0.936), and β2M/Cr. ratio (*p* = 0.573) were found between subgroups.

The β2M level and β2M/Cr. ratio were investigated depending on whether or not the patients received a specific cytostatic drug during the treatment (cyclophosphamide, ifosfamide, cisplatin, and methotrexate); however, no relationships were revealed (*p* > 0.05). Moreover, no correlations between the β2M level and β2M/Cr. ratio, and the cumulative dose of cyclophosphamide, ifosfamide, cisplatin, and methotrexate were found (*p* > 0.05).

Receiving operating curve (ROC) analyses were conducted to determine the diagnostic profile of β2M level and β2M/Cr. ratio in identifying participants with decreased eGFR (<90 mL/min/1.73 m^2^). However, none of them showed any diagnostic value (the AUC for the β2M level was 0.46 and for the β2M/Cr. ratio was 0.43).

The univariable linear regression analysis showed significant correlation between β2M/Cr. ratio and BMI (coef. 14.48, *p* = 0.046, 95%CI 0.24–28.73). Other potential confounding factors such as abdominal radiotherapy, cumulative dose of nephrotoxic cytostatics, age at diagnosis, age at study, eGFR, albumin-to-creatinine ratio, and solitary functioning kidney did not affect the β2M level or β2M/Cr. ratio (Table 4).

## 4. Discussions

The exact etiology of late nephrotoxicity among childhood cancer survivors remains unclear. On the other hand, we know more about treatment-related factors leading to acute kidney injury. Some of these also increase the risk of chronic kidney disease later in life (the most common are described in the introduction). However, there is a population of CCS who had never experienced AKI but who developed CKD many years after treatment. In recent years, much effort has been concentrated on identifying those survivors who are at risk for developing kidney failure during adolescence and adulthood.

The available methods for accessing renal function have many limitations, and, thus, new biomarkers to access early kidney deterioration are needed. There are several urinary markers (highly sensitive and specific) for monitoring drug-induced kidney injury that have been approved in preclinical studies by the European Medicines Agency (EMA) and the Food and Drug Administration (FDA). The best candidates include β2-microglobulin (β2M), kidney injury molecule-1 (KIM-1), neutrophil gelatinase-associated lipocalin (NGAL), clusterin, and trefoil factor-3 (TFF-3) [13,14]. Most studies to date have focused specifically on AKI, and there are limited data on the utility of these biomarkers in late drug-induced nephrotoxicity [15]. In our previous studies on the association between urinary KIM-1 and NGAL levels and late renal toxicity, we showed that CCS had higher levels of both biomarkers, which was related to the cumulative dose of cisplatin and ifosfamide [16,17]. In this prospective cohort study, we focused on β-2-microglobulin and its applicability in accessing renal function in CCS.

In the overall group of CCS, subjects with reduced eGFR (<90 mL/min/1.73 m^2^) accounted for 14.5% and this decreased in subsequent tests performed at least every 3 months. Our results are in accordance with one of the largest available studies conducted on a cohort of 1122 survivors, in which the probability of reduced eGFR among CCS treated with nephrotoxic agents was 6.6% by age 35 years compared to those who had never received such treatment, and eGFR continued to fall with time. The greater risk of nephrotoxicity was observed in survivors given ifosfamide and cisplatin, and with nephrectomy [18,19]. In the present study, no such relationship was noted. This may be explained by the fact that the mean age of our study group was two times lower than in the referred paper and the association is not yet noticeable.

Radiation-associated kidney injury can manifest as decreased eGFR, hypertension, or proteinuria. Dawson et al. reported that 46% of adults who were irradiated with a dose of 20 Gy developed nephropathy [20,21,22]. Similarly, our data showed a significantly higher number of individuals with reduced eGFR in the subset of CCS who were treated with abdominal radiotherapy compared to those who did not receive it, supporting the data on the damaging effects of RT on renal function.

Nowadays, it is increasingly discussed that the evaluation of decline in renal function only with eGFR has many limitations and may misidentify individuals in the early stages of kidney damage as healthy. Moreover, some authors emphasize that serum creatinine level increases when more than forty percent of renal tissue is damaged, which may underestimate the number of patients with early-onset renal injury as assessed by eGFR [23]. For this reason, there is a need for a new, preferably non-invasive biomarker to identify individuals with impaired renal function before it is detectable by standard methods.

The majority of studies conducted so far have looked at serum b2M rather than urine concentrations [5,18]. Most of them pointed to elevated β2M level in patients after chemotherapy with nephrotoxic agents and emphasized its high utility in detecting chemotherapy-induced acute kidney injury [14,24]. On the other hand, there are very limited data on the utility of urinary β2M in diagnosing the onset of CKD associated with anticancer treatment. In the present study, statistically higher level of β2M and β2M/Cr. ratio among CCS were reported. The association between urinary β2M and nephrotoxicity for the first time was described by Tiburcio et al. in a study of 41 CCS [25]. They reported in short-term survivors that urinary β2M level was significantly higher in the study group and was positively correlated with plasma creatinine concentration and negatively correlated with glomerular filtration rate. Similarly, our study confirmed a significantly higher level of urinary β2M in CCS but failed to confirm an association with creatinine level and eGFR value. Only in a small subset of patients with hyperfiltration (7%) was a significantly higher level of β2M found compared to subjects with normal and reduced filtration, which may indicate that increased filtration in the injured kidney enhances β2M excretion and, perhaps, may be a potential tool to assess impaired renal function. However, conclusions from this analysis should be drawn with caution due to the very small sample size. Another study carried out in children previously treated for central nervous system malignancy showed negative correlation between β2M level and eGFR [26]. However, we did not confirm this finding.

To our best knowledge, this is the first clinical study of the association of the urinary β2M among individuals previously treated for acute leukemia (61% of the study group), which has not been investigated so far. These individuals were analyzed separately but did not differ from CCS with other cancer types.

Higher levels of urinary β2M were observed in subjects with a follow-up time of more than 5 years. Interestingly, in the univariable linear regression analysis, significant positive correlations between β2M/Cr. ratio and BMI were observed. These results may suggest that urinary β2M level depends on age and body weight, as postulated by some authors; however, reference values are not available [27,28].

Reviewing the literature, there are many data reporting elevated serum β2M levels at the time of diagnosis in many types of cancer, as well as being a predictor of relapse in some of them [29,30,31]. It is important to note that all participants included in this study were in continuous first remission, and all were carefully screened for relapse and second malignancies.

Furthermore, there are many studies investigating the role of β2M in kidney diseases of different etiology. It has been demonstrated that β2M concentration is elevated among individuals with systemic lupus erythematosus, beta thalassemia major, type 1 diabetes, acute appendicitis, and congenital lower urinary tract obstruction, among others [32,33,34,35,36]. In addition, β2M levels are used as a tumor marker, primarily in monitoring the treatment of multiple myeloma, a cancer that occurs typically in advanced age [37].

Finally, there are several limitations that need to be considered. First, single-center studies may influence the occurrence of bias. Second, the heterogeneity of the study group in terms of different types of cancers made it unfeasible to analyze the effect of some specific treatment protocols (especially very rare tumors). For this reason, we targeted the impact of specific treatments modalities, such as cytostatic agents and radiotherapy. Third, we used the GFR estimated from creatinine level rather than measured by standard techniques such as plasma clearance of iohexol. Another limitation of the study is the non-inclusion of other markers for assessing renal function, such as cystatin C, which is considered to be more resistant to various confounding factors, such as body mass index. The authors did not use Gao’s quadratic equation in addition to Schwartz’s formula when glomerular hyperfiltration was suspected; however, the number of subjects with hyperfiltration was very limited [38].

The strengths of the study include the relatively large number of participants, long follow-up time, and ethnic homogeneity of the group.

Pooling all data, we demonstrated that 14.5% of childhood cancer survivors had reduced eGFR value, and the most adverse factor affecting renal function was abdominal radiotherapy used during the treatment. We also provided new data that this particular population has elevated urinary beta-2-microglobulin level regardless of initial diagnosis. Of the factors tested with potential effects on renal function, only a longer time of follow-up and high BMI were positively correlated with the level of urinary β2M. The results of this study do not explain what urinary β2M level depends on. None of the factors investigated, including type of cancer, cumulative dose of cytostatics used, radiotherapy, nephrectomy, or age at diagnosis significantly affected biomarker levels. It cannot be ruled out that genetic background is also relevant. Furthermore, in light of recent studies demonstrating premature aging of survivors, it is important to note that nephrotoxicity is a multifactorial process involving both environmental and genetic factors [39,40]. Since the process leading to elevated β2M concentration in CCS remains unclear, and its potential use as a biomarker in the assessment of deteriorating renal function is still unexplained, further longitudinal studies on urinary β2M concentration and its potential role as a tool in the assessment of declining renal function are needed.

## Figures and Tables

**Table 1:** Clinical characteristics of the childhood cancer survivors (CCS).

	Total	Male	Female
**Patients (*n*, %)**	165 (100%)	80 (48.5%)	85 (51.5%)
Age at diagnosis (years)	5.31 ± 4.16 4.24 (2.66–7.20)	5.36 ± 4.16 3.82 (2.83–6.9)	5.9 ± 4.67 4.38 (2.34–7.57)
Age at study (years)	13.40 ± 5.96 14.21 (8.62–17.67)	12.25 ± 4.94 12.35 (8.09–16.21)	13.78 ± 5.94 14.5 (9.18–17.42)
Follow-up after treatment (years)	7.01 ± 5.28 6.55 (2.25–10.39)	6.22 ± 5.09 5.51 (2.03–9.25)	7.75 ± 5.27 7.45 (3.83–11.15)
**Diagnosis:**	165 (100%)		
Acute lymphoblastic leukemia	84 (50.9%)	41 (49%)	43 (51%)
Wilms tumor	17 (10.3%)	7 (41%)	10 (59%)
Sarcoma	14 (8.5%)	8 (57%)	6 (43%)
Non-Hodgkin lymphoma	13 (7.9%)	4 (31%)	9 (69%)
Hodgkin lymphoma	10 (6.1%)	4 (40%)	6 (10%)
Neuroblastoma	9 (5.5%)	3 (33%)	6 (64%)
Acute myeloid leukemia	8 (4.8%)	3 (37%)	5 (63%)
Hepatoblastoma	4 (2.4%)	2 (50%)	2 (50%)
Germ tumors	3 (1.8%)	2 (67%)	1 (33%)
Langerhans cell histiocytosis	3 (1.8%)	1 (33%)	2 (67%)
**Chemotherapy:**			
Methotrexate (cumulative dose in mg/m^2^), *n* = 100 (60.6%)	13,373 ± 19,972 8000 (8000–10,000)	14,300 ± 20,059 8000 (8000–20,000)	12,409 ± 20,044 8000 (8000–8000) ^b^
Cumulative corticosteroid ^a^ (dose in mg/m^2^), *n* = 109 (66.1%)	1800 ± 791 1711 (1711–1711) ^b^	1764 ± 763 1711 (1711–1711) ^b^	1838 ± 825 1711 (1711–1748)
Cyclophosphamide (cumulative dose in mg/m^2^), *n* = 113 (68.5%)	3893.36 ± 2448.33 3000 (3000–4000)	3868 ± 235 3000 (3000–3000) ^b^	3919 ± 2557 3000 (3000–3000) ^b^
Ifosfamide (cumulative dose in mg/m^2^), *n* = 14 (8.5%)	74,857 ± 71,888 54,000 (36,000–84,000)	65,500 ± 40,242 54,000 (51,750–78,750)	87,333 ± 104,128 60,000 (18,500–139,500)
Cisplatin (cumulative dose in mg/m^2^), *n* = 16 (9.7%)	430 ± 241.99 400 (240–480)	400 ± 153 400 (240–570)	460 ± 316 400 (240–480)
**Radiotherapy (RT):**	41 (24.8%)	19 (46%)	22 (54%)
Cranial radiotherapy (CRT) (cumulative dose in Gray), *n* = 19 (11.5%)	19.33 ± 14.04 12.0 (12–18)	21.2 ± 16.67 12.0 (12.0–34.2)	17.64 ± 11.85 12.0 (12.0–18.0)
Abdominal radiotherapy (cumulative dose in Gray), *n* = 19 (11.5%)	22.45 ± 10.26 21.0 (19.80–21)	21.98 ± 12.09 19.8 (13.95–21.0)	22.08 ± 9.32 21.0 (19.8–21)
Total body irradiation (TBI) (cumulative dose in Gray), *n* = 5 (3%)	12.0 ± 0.00 12.0 (12.0–12.0) ^b^	12.0 ± 0.0 12.0 (12.0–12.0) ^b^	12.0 ± 0.0 12.0 (12.0–12.0) ^b^
No radiotherapy, *n* = 124 (75.2%)	124 (75.2%)		
**Nephrectomy (unilateral)**	16 (9.7%)	6 (37.5%)	10 (62.5%)
**Hematopoietic stem cell transplantation (HSCT)**	20 (12.1%)	9 (45%)	11 (55%)

Data are given as mean and standard deviation (SD) and median and interquartile range (IQR). There were no statistical differences between males and females; ^a^ calculated as prednisone equivalents; ^b^ most patients received the same dosage of anticancer agents or radiotherapy according to the treatment protocol; therefore, the first and third quartiles did not differ from the median.

**Table 2:** Characteristics of biochemical parameters in childhood cancer survivors.

	Childhood Cancer Survivors *n* = 165	Control Group *n* = 50	*p* Value
Serum creatinine (mg/dL)	0.58 (0.43; 0.75)	0.50 (0.41; 0.63)	0.035
Urine creatinine (mg/L)	120.64 (73.67; 178.52)	89.40 (65.78; 116.44)	0.001
eGFR (mL/min/1.73 m^2^)	117.47 (98.79; 139.87)	118.95 (103.25; 136.02)	0.792
β2M (ng/mL)	14.30 (8.62; 26.10)	8.67 (6.18; 11.48)	<0.001
β2M /cr. (ng/mg cr.)	139.87 (66.41; 305.48)	99.63 (55.34; 162.37)	0.039

eGFR: estimated glomerular filtration rate, β2M: beta-2-microglobulin, cr: creatinine. Data are given as median and interquartile range.

**Table 3:** Characteristics of biochemical parameters according to the time after completion of treatment.

	<5 Years	>5 Years	*p* Value
	*n* = 64	*n* = 101	
Age at study (years)	6.90 (5.42; 11.67)	15.94 (12.99; 19.15)	<0.001
eGFR (mL/min/1.73 m^2^)	127.00 (103.73; 151.96)	112.43 (98.01; 134.98)	0.047
β2M (ng/mL)	10.99 (5.31; 24.50)	16.59 (11.22; 39.82)	<0.0001
β2M /cr. (ng/mg cr.)	110.14 (41.00; 246.49)	151.56 (74.87; 328.48)	0.049

eGFR: estimated glomerular filtration rate, β2M: beta-2-microglobulin, cr: Creatinine. Data are given as median and interquartile range.

**Table 4:** Univariable analysis of the beta-2-microglobulin/creatinine ratio (β2M/Cr. ratio) in childhood cancer survivors.

Variables	Coefficient	*p*
Ifosfamide (cumulative dose)	−17.6	0.120
Cyclophosphamide (cumulative dose)	0.02	0.368
Cisplatin (cumulative dose)	0.14	0.728
Methotrexate (cumulative dose)	−2.92	0.965
Abdominal radiotherapy (yes vs. no)	−89.6	0.368
Age at diagnosis (years)	6.43	0.372
Follow-up time (years)	−0.66	0.922
BMI (kg/m^2^)	14.5	0.046
Hypertension (yes vs. no)	43.2	0.610
Nephrectomy (yes vs. no)	−10.7	0.361
HSCT (yes vs. no)	−45.4	0.642
Diagnosis (leukemia vs. lymphoma vs. solid tumors)	−29.2	0.401

BMI: body mass index, HSCT: hematopoietic stem cell transplantation.

## Data Availability

The data presented in this study are available on request from the corresponding author.
